# Effects of a “silent mentor” initiation ceremony and dissection on medical students’ humanity and learning

**DOI:** 10.1186/s13104-017-2809-0

**Published:** 2017-09-16

**Authors:** Ruei-Jen Chiou, Po-Fang Tsai, Der-Yan Han

**Affiliations:** 1 Department of Anatomy and Cell Biology, School of Medicine, College of Medicine, Taipei Medical University, Taipei, Taiwan; 2 Graduate Institute of Humanities in Medicine, College of Humanities and Social Sciences, Taipei Medical University, Taipei, Taiwan; 3 Section of Liberal Arts, Center for General Education, Taipei Medical University, 250 Wu-Xing Street, Xin-Yi District, Taipei, 11031 Taiwan, ROC

**Keywords:** Attitude towards death, Gross anatomy, “silent mentor” initiation ceremony, Medical compassion

## Abstract

**Objectives:**

Many medical schools in Taiwan have adopted a dignified “silent mentor” initiation ceremony to strengthen student’s medical humanity and increase their learning attitudes. This ceremony consists of introductions of the body donor’s conduct and deeds, wreath-laying, and a tea party. However, few empirical studies have examined the influences of the ceremony and dissection on medical humanity. This study explored if the initiation ceremony and the course can help students care more about others, develop more positive attitudes toward death, improve learning effectiveness in the course, and decrease negative emotions the first time they see a cadaver.

**Methods:**

The Attitudes Towards Death and Love and Care subscales of the life attitude inventory, Learning Effectiveness of Gross Anatomy Laboratory Scale (LEGALS), and Emotional Reactions Towards Cadavers Scale were adopted to examine differences before (T1) and after (T2) medical students attended an initiation ceremony at a university in northern Taiwan. Whether these effects lasted to the end of the semester (T3) was also tested.

**Results:**

After the ceremony, students’ attitudes towards death increased, negative emotions towards cadavers decreased, but love and care and the LEGALS did not significantly change. Data from T3 showed a similar pattern, but high-level emotions (e.g., being respected, cherished, and grateful) and the LEGALS were significantly higher than those at T1.

**Discussion:**

The initiation ceremony, which showed a body donor’s deeds and attitudes toward life and death when they were alive, could help medical students gain more mature attitudes towards death and decreased negative emotions. Learning between T2 and T3 might have caused significant changes in high-level emotions and the LEGALS at T3. Arranging reflective writing with guided discussion by a teacher before and after the ceremony is highly recommended.

## Background

Since the second half of the twentieth century, the curriculum of medical humanities has gained in importance as a part of medical education. To change the narrow perspective which views human subjects as treatment objects, these curricula are expected to provide a function that re-humanizes the biomedical paradigm, in terms of perspective taking, critical thinking, and language usage [1, 2]. Medical education in Taiwan also echoes this trend, by trying to strengthen student’s medical humanity with local Taiwanese features.

There have been some reflections on medical humanities education in Taiwan since the 1990s: most courses are confined to the first two school years of medical students and conducted under the concept of general education, but students are not interested in these courses [3, 4]. This conventional practice, on the one hand, truncates medical students’ learning processes into two parts, basic medical science and medical humanities. On the other hand, it also causes difficulties in distributing teaching resources in curricular design and under-integration between faculty members in biomedicine and those in humanities, and even obstructs interactions between teaching and learning [4, 5]. In order to overcome those shortcomings and improve medical humanities education, the teaching faculty has to create synergy between specialties and generalities, biomedicine and humanities [4–7].

To date, studies on learning attitudes and motives of medical students in medical humanities indicated that teachers have to face the reality that students’ focus on the acquisition of professional practical skills diminishes their interest in non-practical courses, even if those courses are personally enriching [8–11]. Therefore, it is necessary to include the spirit and training of medical humanities into professional biomedical curricula to avoid the decoupling separation of “pre-medical, basic-medical, and clinical medical” curricular design. One feasible solution is to reform the curriculum itself, shifting from “medical humanities in lecture” to “medical humanities in practice.”

As far as professional courses that medical students take are concerned, the Gross Anatomy Laboratory occupies a crucial position during their learning years. The course Gross Anatomy Laboratory runs in the autumn of students’ 3rd school year. Around 20 students use one cadaver to learn dissection in a semester. For most students, it is the first time they see and touch a cadaver. In these years, we found that some students felt frightened, others felt respected, and still others felt excited. To release student’s emotions and increase their medical humanity, we hold a “silent mentor” initiation ceremony before the first actual dissection begins. Teachers explain how to dissect in the classroom before a dissection actually occurs each time. After that, students enter the dissection room to dissect and find out about body structures which were mentioned in the lecture.

The Gross Anatomy Laboratory is not only an important “rite of passage” in the formation of a medical student’s professional learning and identity, but also the first influential moment in which they have to face the issues of death, human life, and other ethical problems [12–14]. Hence, it is a good chance to integrate contents of medical humanities into the practice of gross anatomy. Many medical schools in Taiwan have incorporated a dignified ceremony to develop a humanitarian spirit and improve both the learning efficacy of students during their university years and the vocational commitment in their clinical years.

Rites, symbolic activities held at a certain period of time and in a particular venue, exert an important influence which allows participants to express their feelings or enter the same emotion [15, 16]. Among them, a “rite of passage” is a concept widely used in anthropology, which involves a series of identity changes from a person’s birth to death [pp. 94–130, 17, pp. 11–14, 18]. The initiation ceremony before the dissections is based on the concept of a “rite of passage.” After we called body donors “silent mentors,” the identity of the donor changes from an anonymous cadaver specimen to a teacher who teaches students knowledge of human structures [14].

The ceremony commends silent mentors for their sacrifice and devotion to the continuation of life, and it also thanks relatives of the donor for their support. In the ceremony, death is no longer a fearsome subject, and many students vow that they will study harder and carry out their duties diligently while they are students in school and after they become doctors in the future. They promise not to let the silent mentors or their families down [14].

However, few empirical studies have demonstrated the positive influences of initiation ceremony on medical students. Some scholars indicated that the negative reactions—anxiety, fear, or depression—appeared when medical students encounter human cadaver in their gross anatomy class. Those emotions happen constantly not only in different major medical programs like medical, dentistry and pharmacy, but also in the national contexts, such as UK, Romania, Spain and Hungary [19–25]. Although researchers mainly focus on the negative emotions, the coping strategies of medical students to overcome the anxiety or fear were also mentioned, such as repeated or gradual exposure, peer discussion, and short documentary video [19, 25–27]. In addition to these means to reduce the negative emotions, some scholars had found that gratitude ceremony or cadaver naming can be act by the way that re-humanize medical students’ response to the dissection of a human corpse [28, 29].

The present study tries to adopt an appropriate inventory of attitudes toward life and death, and develops scales to assess the learning effectiveness of the Gross Anatomy Laboratory course and emotions when students see a cadaver for the first time in order to realize the effects of the initiation ceremony and the course. We suggest that the initiation ceremony impacts both the emotions and cognition of students. It decreases negative emotions such as fear and worry, improves students’ attitudes towards life and death, and strengthens their devotion to the Gross Anatomy Laboratory course and their medical career.

Since it is inappropriate to conduct a randomized controlled trial in educational circumstances, we adopted repeated measures over 7 days before and after the ceremony, with a follow-up at the end of the semester. We hypothesized that the initiation ceremony would influence students’ attitudes towards death, care for others, emotional reactions towards cadavers, and learning effectiveness of the course.

## Methods

### Participants

Participants consisted of 158 third year medical students of a medical university in northern Taiwan. The sample included all the students who were willing to participate in this study in that term. Their average age was 20.97 ± 1.53 years. There were 96 males and 62 females.

According to research ethics, participants had the right to dropout anytime without any justification. Some participants quitted this study after they finished the first questionnaire. Eventually, there were 153 participants right after the ceremony and 137 participants in the followup session. The demographic variables that students who joined and did not joined the 2nd or 3rd wave data collection were not different.

### Material and instruments

#### Life attitude inventory (LAI)

To measure life attitudes of college students, Pan and Hsieh developed the LAI based on the concepts of life from the existentialists Jean-Pau1 Sartre (1905–1980) and Rollo May (1909–1994), humanistic psychologist Carl Rogers (1902–1987), and the founder of Logotherapy, Viktor Frankl (1905–1997) [30]. This inventory is rated on a 7-point Likert-type scale, with a higher score representing a more mature attitude towards life and death. The original Cronbach’s α was .93, and the 4-week test–retest reliability was .91. Results of factor analyses and hierarchical clustering analyses proved the six-dimension construct. To measure medical students’ relevant attitudes toward life and death, we adopted only two subscales. The first one, Love and Care, assesses interpersonal attitude towards other people and behaviors adopted when they interact with people (e.g., “I am willing to spend time to accompany people who need comfort”, and “I usually approach others actively and accept them”). A higher score represents a better attitude to giving others love unconditionally. The second one, Attitudes Towards Death, assesses anticipation, attitudes, and actions toward death (e.g., “I want to explore issues relevant to death”, and “I can accept death calmly, even though I can’t anticipate when it will come.”). A higher score represents a more mature attitude towards death.

#### Learning Effectiveness of the Gross Anatomy Laboratory Scale (LEGALS)

To evaluate students’ overall learning effectiveness, the LEGALS measures the extent to which medical students become appreciative, cherishing, compassionate, aware of life and death, and willing to do their best to learn when taking the course of Gross Anatomy Laboratory (e.g., “This course makes me have more medical humanity virtue”, and “This course strengthens my motivation to become a good physician”). Based on students’ descriptions after visiting family members of a donated cadaver, and observations from faculty members for many years, we listed 13 items to ascertain students’ possible changes. The LEGALS is rated on a 7-point Likert-type scale (1: strongly disagree to 7: strongly agree). Item analyses showed that no items should be deleted to largely increase the internal consistency, so all items were retained. Cronbach’s α of the LEGALS was .96. A higher score represents a better humanistic learning effectiveness from the course of Gross Anatomy Laboratory.

#### Emotional Reactions Towards Cadavers Scale (ERTCS)

Based on students’ descriptions after the first time they saw a cadaver and emotional reactions observed by leaders of the course, we listed 25 main emotions to measure students’ emotional reactions after they see a cadaver. The ERTCS is rated on a 7-point Likert-type scale (1: strongly disagree to 7: strongly agree), and Cronbach’s α of the scale was .84. Because of the variety of emotional reactions, an exploratory factor analysis with the method of maximum-likelihood factoring was utilized and yielded a satisfactory three-factor structure. PROMAX rotation was further adopted, and 49.47% of the total variance was accounted for by the three factors, negative emotions (e.g., being fearful, terrified, and spooky), high-level emotions (e.g., being respected, cherished, and grateful), and excited emotions (e.g., being fully expected, happy, and curious). Respective Cronbach’s α values of the three factors were .88, .90, and .84. A higher score represents a stronger feeling when students see a cadaver.

#### Demographic variables

We collected student’s basic demographic variables including gender, age, religious belief, and family closeness.

### Content of the Gross Anatomy Laboratory course

In our course of Gross Anatomy Laboratory, students were divided into 8 groups, and each group further divided into 5 divisions. The whole body was divided into 5 dissection regions including head region, neck and thoracic region, upper limb region, abdominal and pelvic region and lower limb region. Each division of students was responsible for one dissection region, and each dissection region was supervised by one teacher. Before dissection, lecturers explained how to dissect the region and reminded students that some structures needed attention in the classroom. When dissection started, students gathered around the dissection table and greeted to the silent mentor. During the dissection, teachers would walk around the dissection tables and give some advices to students who had difficulties. At the end of the dissection, students also gathered around the table and said “thank you” to the silent mentors. At the end of the semester, after the final term examination, the organs were replaced to the original position, the incised skin was sutured. The whole body restored to the former appearance. The Department of Anatomy and Cell Biology would choose a day to place the cadaver to the coffin. The students saw the silent mentors off to the crematorium to cremate until the end. The whole gross anatomy dissection course completed.

### Procedures

#### Research procedures

Third-year medical students filled out a battery of questionnaires over 7 days before (T1, on November 4) and after (T2, on November 11) the “silent mentor” initiation ceremony in 2015. The first time students saw the silent mentor was about 3 days before the ceremony. After the ceremony, the course formally began. To evaluate changes at the end of the semester, they filled out the battery again in January 2016 (T3).

#### Ceremony procedures

Participants of the initiation ceremony included family members of the silent mentors, third-year medical students, and faculty members of Anatomy and Cell Biology. In the ceremony, each group of students openly introduced and commemorated the silent mentor’s conduct and deeds, and then laid a wreath. The contents of introductions were from a home visitation carried out between students and the silent mentor’s family members before the ceremony, which included stories during their lifetime, relationships with family members and friends, and reasons for being a body donor. After that, a tea party was held to allow students and family members to share feelings with each other. In the end, students accompanied the family members to the university gate and ended the initiation ceremony.

### Data processing

Paired *t*-tests were conducted to compare students’ attitudes towards death, care for others, emotional reactions towards cadavers, and learning effectiveness before (T1) and after (T2) the ceremony, and the differences between them and the 3-month followup (T3). Statistic software SPSS 20.0 was utilized to perform the statistical tests.

## Results

The descriptive statistic and *t*-tests results are shown in Tables 1 and 2 respectively. The results of paired *t*-tests showed that the level of Attitudes Towards Death of participants increased after the ceremony (T1 to T2), *t* = −4.12, *df* = 151, *p* < .001. The result suggested that participants had more reflection of death after the ceremony.

**Table 1:** Numbers and percentages of participants’ demographic variables

Variables	T1		T2		T3		*F/χ* ^2^	*df*	*p*
	*n*	%	*n*	%	*n*	%			
Total	158	100	153	96.8	137	86.7			
Age (mean ± SD)^a^	20.97 ± 1.53		20.99 ± 1.54		21.09 ± 1.38		*F* = 20.38	1	<.05
Gender									
Male	96	60.8	92	60.1	83	60.6	*χ* ^2^ = .014	2	.993
Female	62	39.2	61	39.9	54	39.4			
Religious belief									
Yes	69	43.67	66	56.2	60	43.8	*χ* ^2^ = .510	2	.972
No	87	55.06	86	43.1	75	54.7			
No answer	2	1.27	1	7	2	1.5			
Family closeness									
Very good	96	60.8	93	60.8	83	60.6	*χ* ^2^ = .622	6	.996
Good	44	27.8	43	28.1	41	29.9			
Middle	15	9.5	14	9.2	10	7.3			
Poor	3	1.9	3	2.0	3	2.2			
Very poor	0	0	0	0	0	0			

^a^Age among the three time points were different merely because the participants were aging

**Table 2:** Paired *t* test values before and after the initiation ceremony

	Pre-ceremony (T1)	Post-ceremony (T2)	Follow-up (T3)	*t* ^T1–T2^	*df*	*p*	*t* ^T2–T3^	*df*	*p*	*t* ^T1–T3^	*df*	*p*
Love and care	5.54 ± .82	5.46 ± .91	5.49 ± .92	1.35	152	.178	−.19	132	.851	1.17	136	.242
Attitude towards death	4.77 ± .54	5.04 ± .85	5.10 ± .75	−4.12	151	.000	−.60	129	.548	−6.28	131	.000
Negative emotions	2.98 ± .94	2.52 ± .95	2.34 ± 1.04	5.62	151	.000	1.81	132	.072	6.71	135	.000
High-level emotions	5.52 ± 1.07	5.40 ± 1.12	5.87 ± 1.32	1.35	151	.180	−3.34	132	.001	−3.00	135	.003
Excited emotions	3.96 ± 1.02	4.09 ± .90	4.09 ± 1.04	−1.49	151	.138	.27	132	.784	−.74	135	.462
LEGALS^a^	5.65 ± 1.04	5.53 ± 1.10	5.84 ± .95	1.51	151	.132	−2.51	129	.013	−2.11	131	.037

^a^Learning Effectiveness of Gross Anatomy Laboratory Scale

The participants’ level of negative emotions toward cadavers decreased after the ceremony (T1 to T2), *t* = 5.62, *df* = 151, *p* < .001. It suggested that the participants felt less negatively to the cadavers after the ceremony. In short, the results partially supported our hypothesis.

The LEGALS and Love and Care, however, showed no significant difference before and after the ceremony (for LEGALS: *t* = 1.51, *df* = 151, *p* = .132; for Love and Care: *t* = 1.35, *df* = 152, *p* = .178).

We further examined whether participants’ changes of attitude and emotion would last for 3 months after the ceremony. The results of paired *t*-tests showed that the level of Attitudes Towards Death remained increased (*t* = −6.28, *df* = 131, *p* < .001), and the level of negative emotions toward cadavers remained decreased 3 months after the ceremony (*t* = 6.71, *df* = 135, *p* < .001).

In addition, participants’ levels of high-level emotions and LEGALS significantly increased after 3 months (for high-level emotions: *t* = −3.00, *df* = 135, *p* < .001; for LEGALS: *t* = −2.11, *df* = 131, *p* = .037).

## Discussion

In our past experiences, we found out the students are more responsible for the course, and also sensed others’ deeper expectations for them after home visit and initiation ceremony. However, there was no research to investigate the exact changes. From results of the present study, we knew that the score of Attitudes Towards Death increased, and the score of negative emotions toward cadaver decreased. In addition, high-level emotions and LEGALS increased at the end of the semester. It showed that these activities were effective in increasing student’s medical humanities.

Changes in attitudes towards death before and after the initiation ceremony might have come from the silent mentor’s lifetime conduct and deeds and their keen dedication at the end of their lives. Donating their body was most silent mentors’ last wish, which makes a life without regrets, and they were inclined to tell their families to definitely carry out this wish. The lifetime conducts of many silent mentors can be a learning model for students both in manners dealing with people and matters and attitudes towards difficulties. These sections of life move students a lot, and they finally understand that the fulfillment of wishes and pursuit of last aspirations are examples worth learning. Silent mentors were inclined to think about how they could devote all of themselves to society even when they were facing death, which leaves an impression on students that silent mentors were keen on dedication even at the end of life, reminding students that death is not a horrible thing. Silent mentors played the first role allowing most students who lacked experiences facing death to reflect on this issue. Close contact with a silent mentor’s family members also made students cherish their own lives. The initiation ceremony remarkably increased students’ attitudes towards death.

According to the literature, some students showed negative emotions at the very beginning of their practice in the course, Gross Anatomy Laboratory [19–25]. While most researchers had tried to prove different ways of decreasing students negative emotions, their research inclined to view students’ coping strategies as impassive mechanisms of overcoming their anxiety and fear [19, 25–27]. Few scholars attempting to change this conventional viewpoint and thus valuing the active and positive impact of initiation ceremony [28, 29]. This study filled the gap of current research about the negative emotions and coping strategies with a focus on initiation ceremony.

Our results showed that the negative emotions were weak, and initiation ceremony further reduced those weak uncomfortable feelings. The face-to-face home visits arranged for students and the family of the silent mentor before the initiation ceremony contributed to further changing the relationships between students and the silent mentor. Before the visit, silent mentors were just strangers to the students; after the visit, they became elders or friends next door. Distances between them were reduced, and negative emotions were also released. The initiation ceremony turned silent mentors into teachers who taught students the human body’s structure, directions of the muscles and blood vessels, and the distribution of nerves within their bodies. As a result, negative feelings were reduced.

At the time the new semester began, students realized the sacrifice and dedication of the silent mentors from the descriptions of senior students and teachers who showed their respect and affection for the silent mentors, so that the score of high-level emotions exceeded other emotions at T1. According to our findings, high-level emotions were significantly elevated from T2 to T3 (*t* = 3.34, *df* = 132, *p* < .001). We believe that interactions between students and the silent mentors after the ceremony enhanced feelings of respect and gratitude. As students learned more from their silent mentors during the semester, they experienced more high-level emotions. The changing pattern of the LEGALS was similar.

Results of T2 and T3 showed that the score of Love and Care did not significantly change. This might have been because the initiation ceremony itself did not sufficiently influence student’s interpersonal attitudes. A study showed that educators read and respond to students’ reflective writing on experiences in the gross anatomy lab helps the processes of students’ professional formation [23]. Reflective writing with guided discussion by a teacher is suggested to be a good teaching technique in the future to improve student’s humanity.

There are some limitations of the study. (1) Because of the conditions of an educational circumstance, it was difficult to design a completely randomized control trial to demonstrate differences between students who joined the initiation ceremony and those who did not. (2) Although questionnaires were collected at the end of the semester, it was unknown whether the significant results at T3 came from post effects of the ceremony, learning from the silent mentors, or even other courses. In the same semester, students also took the course, Introduction to Social Medical Sciences, which is related to medical humanity development. (3) The home visit is an important activity accompanying the Gross Anatomy Laboratory in our study. It was hard to control if senior students or teachers described the home visit to the students on informal occasions.

In the future, we will try to design more delicate procedure of the home visit and the ceremony, and make students engage more in these activities. The home visit, ceremony and gross anatomy lab all play a role in cultivate students’ medical humanities. There should be other relative courses to extend these issues such as life and death and how to deal with grief, which are very important to these students who will be doctors in the near future. It is also what we plan to do the next step.

## Conclusion

The “silent mentor” initiation ceremony could help medical students improve their attitudes towards death, and had a role in soothing negative emotions. Together with the course Gross Anatomy Laboratory, it might have increased their performance on practice attitudes and medical humanity virtues in the future. There are many medical schools and universities that adopt initiation ceremonies in Taiwan nowadays [13]; however, whether places other than Taiwan or students other than those in medical school could also benefit from these ceremonies or humanity courses still needs to be examined in future studies.
